# Dataset describing ethanol and 1,2-propanediol production by a stenothermal moderately thermophilic anaerobe, *Clostridium* strain AK1

**DOI:** 10.1016/j.dib.2018.08.088

**Published:** 2018-08-30

**Authors:** Eva Maria Ingvadottir, Sean Michael Scully, Johann Orlygsson

**Affiliations:** University of Akureyri, Faculty of Natural Resource Sciences, Borgir 2 v/Norðurslóð, 600 Akureyri, Iceland

**Keywords:** Propylene glycol, Deoxy sugar, Methylpentose, Chiral alcohol, Moderate thermophile

## Abstract

The dataset details the fermentation of D-glucose, L-rhamnose, and L-fucose and their end-product formation by the moderate thermophile *Clostridium* strain AK1 (DSM 18778) as related to the work described in “Propanediol from L-rhamnose using the moderately thermophilic *Clostridium* strain AK1” [1]. The influence of culture conditions on end product formation from D-glucose and L-rhamnose by AK1 was investigated in batch culture. Strain AK1 was cultivated at initial substrate concentrations varying from 0 to 60 mM and initial pH values varying from 4.5 to 8.5. Additionally different cultivation temperatures (30–65 °C), the influence of liquid-gas phase ratio as well as different phosphate concentrations on growth were investigated.

**Specifications Table**

**Table:** 

Subject area	Biology
More specific subject area	Microbiology
Type of data	Table
How data was acquired	GC-FID, Perkin Elmer Clarus 580
	GC-TCD, Perkin Elmer Autosystem XL
	UV-Visible Spectroscopy, Bioscreen C (GrowthCurves Ltd, Finland) and Shimadzu UV-1800 UV-Visible Spectrometer
Data format	Raw
Experimental factors	Substrate, growth temperature, initial substrate concentration, initial pH, liquid-gas phase ratio, initial phosphate concentration
Experimental features	Data shows fermentation products from D-glucose, L-rhamnose, and L-fucose under different culture parameters. Major end products being ethanol (from glucose) and 1,2-PD from L-rhamnose. L-fucose was not fermented.
Data source location	University of Akureyri, Akureyri, Iceland
Data accessibility	Data is included in this article
Related research article	Ingvadottir et al. [1]

**Value of the data**

• The data presents ethanol and 1,2-propanediol fermentation from a stenothermal moderately thermophilic anaerobe isolated from a geothermal feature in Iceland.
• This data set could be of value for comparing fermentation yields and the impact of key culture parameters on ethanol and 1,2-propanediol producing microorganisms.
• Could be used as a reference organism for studying an overlooked environmental niche (moderate temperature hot springs).

## 1 Data

*Clostridium* strain AK1 ferments D-glucose and L-rhamnose with ethanol and 1,2-propanediol (1,2-PD) being the major end-products, respectively (Table 1). Table 2 shows the influence of temperature on the fermentation of D-glucose and L-rhamnose while the effect of pH from pH 4.8 to 7.8 is shown in Table 3. The impact of liquid-gas phase ratio in culture bottles on D-glucose and L-rhamnose fermentation is summarized in Table 4. D-Glucose and L-rhamnose degradation on various initial phosphate concentrations resulted in the same end-product formation as before as summarized in Table 5.

**Table 1:** Effect of initial substrate concentration (10 to 60 mM) D-glucose, L-fucose, and L-rhamnose by *Clostridium* strain AK1 (DSM 18778). Values represent the average±standard deviation of triplicates.

Substrate (mM)	Sugar consumed (%)	Analyte (mmol L^-1^)										
		Ethanol	Acetate	Butyrate	1.2-PD	Lactate	H_2_	OD_600 nm_	pH	Ethanol Yield (%)	1.2-PD Yield (%)	Carbon balance (%)
Control (0)	ND	2.95±0.10	1.82±0.10	< 0.20	< 0.20	0.20±0.10	1.80±0.10	0.09±0.01	6.9±0.1	ND	ND	ND
D-Glucose (10)	88.9±1.1	11.00±0.40	6.10±0.64	0.28±0.07	< 0.20	0.45±0.20	8.29±0.23	0.26±0.01	6.8±0.1	55.0	0.0	104.6
(20)	94.9±2.4	23.71±3.23	11.20±1.93	< 0.20	< 0.20	1.48±0.36	10.49±3.57	0.33±0.02	6.6±0.2	59.2	0.0	97.5
(40)	89.6±3.3	38.66±1.19	10.48±0.29	«0.20	< 0.20	2.72±1.44	20.45±0.70	0.35±0.01	5.8±0.1	48.3	0.0	73.9
(60)	47.0±3.4	36.69±0.81	9.71±0.28	< 0.20	< 0.20	6.27±1.04	20.30±1.13	0.37±0.02	5.8±0.1	30.6	0.0	96.6
L-Fucose (10)	0.0±0.0	2.11±0.22	1.34±0.26	< 0.20	< 0.20	< 0.20	0.93±0.37	0.08±0.02	6.9±0.1	ND	0.0	ND
(20)	0.0±0.0	1.04±0.17	0.53±0.34	< 0.20	< 0.20	< 0.20	0.64±0.24	0.07±0.01	6.9±0.1	ND	0.0	ND
(40)	ND	ND	ND	ND	ND	ND	ND	ND	ND	0.0	ND	ND
(60)	ND	ND	ND	ND	ND	ND	ND	ND	ND	0.0	ND	ND
L-Rhamnose (10)	80.1±2.2	2.20±0.03	3.50±0.32	< 0.20	6.60±0.75	< 0.20	1.21±0.23	0.17±0.01	7.1±0.2	22.0	66.0	79.0
(20)	74.9±0.8	2.03±0.07	6.59±0.04	0.83±0.12	12.20±0.37	< 0.20	2.40±0.15	0.19±0.02	6.9±0.1	10.2	61.0	82.8
(40)	59.9±1.2	1.90±0.15	17.68±0.35	1.05±0.21	22.13±3.21	< 0.20	4.71±0.42	0.30±0.01	6.2±0.1	4.8	55.3	95.4
(60)	34.9±2.8	1.54±0.21	12.80±1.01	0.80±1.01	21.62±2.88	< 0.20	3.15±0.72	0.27±0.04	6.0±0.3	2.6	36.0	82.5

**Table 2:** Effect of cultivation temperature on fermentation of selected sugar (20 mM) by *Clostridium* strain AK1 (DSM 18778). Values represent the average of triplicates with standard deviation shown as error bars.

Substrate	T (°C)	Sugar consumed (%)	Analyte (mmol L^-1^)												
			Ethanol	Acetate	Butyrate	1,2-PD	Lactate	H_2_	OD_600 nm_		pH	Ethanol Yield (%)		1,2-PD Yield (%)	Carbon balance (%)
D-Glucose	30	37.0±2.1	6.89±1.31	5.53±0.87	< 0.20	< 0.20	0.10±0.10	0.22±0.28	0.13±0.05	6.8±0.1		17.2		0.0	87.5
D-Glucose	35	35.1±3.8	7.10±0.59	4.21±0.14	< 0.20	< 0.20	0.40±0.10	0.05±0.03	0.11±0.06	7.0±0.2		17.8		0.0	85.2
D-Glucose	40	34.1±1.3	6.31±0.49	4.48±0.34	< 0.20	< 0.20	0.50±0.10	0.59±0.16	0.23±0.03	6.9±0.1		21.4		0.0	84.7
D-Glucose	45	33.3±0.9	8.54±2.57	4.56±0.71	< 0.20	< 0.20	0.60±0.23	2.87±0.73	0.24±0.05	6.8±0.1		21.4		0.0	99.4
D-Glucose	50	96.0±1.3	29.40±2.67	9.10±0.47	< 0.20	< 0.20	1.55±0.17	9.65±0.67	0.20±0.02	6.6±0.2		73.5		0.0	104.8
D-Glucose	55	93.9±2.2	27.56±2.02	9.40±1.14	< 0.20	< 0.20	1.87±0.28	10.04±0.67	0.36±0.02	6.7±0.2		68.9		0.0	103.8
D-Glucose	60	0.0±0.0	1.35±0.23	1.03±0.80	< 0.20	< 0.20	0.43±0.10	0.02±0.00	0.12±0.01	6.8±0.1		< 0.5		0.0	ND
D-Glucose	65	0.0±0.0	1.23±0.06	1.16±0.03	< 0.20	< 0.20	0.30±0.10	0.19±0.27	0.12±0.01	6.7±0.0		< 0.5		0.0	ND
L-Rhamnose	30	0.0±0.0	1.97±0.08	2.32±0.11	0.67±0.76	0.29±0.25	< 0.20	0.83±0.10	0.07±0.01	6.9±0.1		9.9		1.5	ND
L-Rhamnose	35	15.5±2.3	2.08±0.23	2.02±0.15	0.54±0.11	0.20±0.10	< 0.20	0.36±0.14	0.11±0.03	6.9±0.0		10.4		1.0	86.8
L-Rhamnose	40	19.0±1.3	1.97±0.01	2.21±0.05	0.87±0.30	0.97±0.16	< 0.20	0.82±0.08	0.15±0.01	6.9±0.1		19.7		4.9	90.6
L-Rhamnose	45	27.0±2.2	2.19±0.43	2.00±0.30	2.02±0.29	2.83±0.70	< 0.20	2.16±0.30	0.14±0.01	6.8±0.1		21.9		14.2	102.4
L-Rhamnose	50	66.0±2.8	3.29±0.16	8.89±0.28	1.54±0.35	9.72±0.71	< 0.20	2.81±0.35	0.13±0.01	6.9±0.2		32.9		48.6	94.6
L-Rhamnose	55	60.5±1.2	3.29±0.24	10.04±1.76	1.12±0.15	7.22±0.63	< 0.20	2.39±0.54	0.13±0.02	6.8±0.1		32.9		36.1	94.2
L-Rhamnose	60	0.0±0.0	1.43±0.19	0.80±0.20	1.83±0.57	0.32±0.24	< 0.20	0.02±0.00	0.13±0.01	7.0±0.1		10.4		1.6	ND
L-Rhamnose	65	0.0±0.0	1.35±0.04	0.55±0.02	0.48±0.06	< 0.20	< 0.20	0.03±0.01	0.12±0.02	7.2±0.1		13.5		0.0	ND

**Table 3:** Effect of initial pH on fermentation of selected sugar (20 mM) by *Clostridium* strain AK1 (DSM 18778). Values represent the average of triplicates with standard deviation shown as error bars.

Substrate	Initial pH	Sugar consumed (%)	Analyte (mmol L^-1^)												
			Ethanol	Acetate	Butyrate	1,2-PD	Lactate	H_2_	OD_600 nm_	pH		Ethanol Yield (%)		1,2-PD Yield (%)	Carbon balance (%)
D-Glucose	4.5	0.0±0.0	2.30±0.30	1.10±0.20	< 0.20	< 0.20	0.33±0.04	1.40±0.02	0.09±0.03	4.2±0.3		5.8		0.0	ND
D-Glucose	4.8	77.0±0.6	16.51±2.06	11.03±1.49	< 0.20	< 0.20	1.10±0.10	11.86±3.70	0.50±0.03	4.4±0.0		41.3		0.0	94.3
D-Glucose	5.1	72.1±0.9	16.19±0.66	10.42±0.40	< 0.20	< 0.20	1.40±0.13	9.29±0.09	0.49±0.02	4.7±0.2		65.4		0.0	98.6
D-Glucose	5.5	90.1±1.3	26.15±2.20	12.12±0.12	< 0.20	< 0.20	1.30±0.18	8.83±1.09	0.46±0.02	5.1±0.1		65.4		0.0	104.0
D-Glucose	5.9	90.1±1.2	21.82±1.48	12.95±0.40	< 0.20	< 0.20	1.90±0.21	9.55±1.56	0.44±0.03	5.6±0.1		54.5		0.0	101.8
D-Glucose	6.7	97.7±0.4	34.62±1.60	14.15±1.28	< 0.20	< 0.20	1.44±0.30	8.30±1.25	0.34±0.02	6.6±0.2		86.6		0.0	108.9
D-Glucose	7.2	92.7±1.5	24.34±2.77	12.81±0.72	0.25±0.01	< 0.20	1.40±0.25	8.91±0.13	0.37±0.02	6.8±0.1		60.9		0.0	105.6
D-Glucose	7.8	93.8±1.4	27.45±1.50	14.68±1.10	0.34±0.00	< 0.20	1.00±0.10	8.43±0.50	0.33±0.06	7.5±0.1		68.6		0.0	105.5
D-Glucose	8.5	0.0±0.0	2.10±0.22	1.00±0.08	< 0.20	< 0.20	0.20±0.10	1.20±0.04	0.11±0.03	8.3±0.2		5.3		0.0	ND
L-Rhamnose	4.5	0.0±0.0	0.40±0.06	1.20±0.03	0.10±0.01	0.80±0.10	< 0.20	0.20±0.07	0.07±0.04	4.1±0.1		2.0		4.0	ND
L-Rhamnose	4.8	52.1±1.9	1.74±0.19	10.13±0.82	1.60±0.23	6.46±0.84	< 0.20	3.01±0.73	0.14±0.06	4.5±0.2		8.7		32.3	103.5
L-Rhamnose	5.1	66.0±0.7	3.07±0.45	10.92±0.53	2.10±0.22	7.63±0.84	< 0.20	3.05±0.70	0.14±0.02	4.8±0.1		15.4		38.2	97.8
L-Rhamnose	5.5	77.5±0.5	0.94±0.97	12.99±1.54	2.00±0.30	9.22±1.46	< 0.20	3.22±0.86	0.16±0.03	5.1±0.2		4.7		46.1	87.6
L-Rhamnose	5.9	71.5±1.1	1.24±0.13	10.72±0.25	1.90±0.23	8.42±1.57	< 0.20	2.49±0.76	0.13±0.02	5.4±0.2		6.2		42.1	84.5
L-Rhamnose	6.7	77.0±1.5	2.22±0.46	15.10±0.84	1.30±0.26	10.38±1.19	< 0.20	3.48±0.84	0.16±0.04	6.3±0.1		11.1		51.9	98.4
L-Rhamnose	7.2	61.5±2.2	1.33±0.14	10.10±0.30	1.00±0.30	8.49±0.16	< 0.20	2.73±0.21	0.19±0.01	6.9±0.2		6.7		42.5	89.1
L-Rhamnose	7.8	56.1±3.0	0.94±0.30	10.60±1.99	1.50±0.22	7.54±1.79	< 0.20	2.32±0.55	0.21±0.01	7.3±0.1		4.7		37.7	98.6
L-Rhamnose	8.5	0.9±0.3	0.40±0.00	1.00±0.05	0.20±0.05	0.70±0.03	< 0.20	0.23±0.02	0.98±0.06	8.4±0.1		2.0		3.5	ND

**Table 4:** Effect of liquid-gas phase ratio on fermentation of selected sugar (20 mM) by *Clostridium* strain AK1 (DSM 18778). Values represent the average of triplicates with standard deviation shown as error bars.

Substrate	L-G ratio	Sugar consumed (%)	Analyte (mmol L^-1^)										
			Ethanol	Acetate	Butyrate	1,2-PD	Lactate	H_2_	OD_600 nm_	pH	Ethanol Yield (%)	1,2-PD Yield (%)	Carbon balance (%)
D-Glucose	0.09	96.1±1.5	22.20±3.02	14.10±1.69	0.25±0.10	< 0.20	1.71±0.65	2.40±0.20	0.37±0.01	6.4±0.2	55.5	0.0	101.8
D-Glucose	0.34	97.3±0.8	26.42±4.81	11.50±0.74	< 0.20	< 0.20	2.63±0.13	6.40±1.23	0.35±0.01	6.3±0.1	66.1	0.0	106.1
D-Glucose	1.00	97.0±0.3	24.04±2.60	11.90±0.93	< 0.20	< 0.20	4.46±0.69	18.51±1.75	0.35±0.01	6.4±0.1	60.1	0.0	106.3
D-Glucose	2.12	96.0±1.5	26.11±1.33	9.20±0.36	< 0.20	< 0.20	1.34±0.47	12.89±0.37	0.34±0.02	6.1±0.1	65.3	0.0	97.5
D-Glucose	5.62	95.0±1.3	26.92±2.56	10.00±0.49	< 0.20	< 0.20	0.79±0.18	41.62±1.21	0.34±0.01	6.2±0.2	67.3	0.0	101.3
L-Rhamnose	0.09	67.5±1.4	1.23±0.62	9.23±2.71	1.83±0.65	12.19±1.96	< 0.20	0.34±0.07	0.19±0.01	6.8±0.1	6.2	61.0	97.4
L-Rhamnose	0.34	53.0±3.4	3.65±0.44	5.87±0.38	1.42±0.99	6.16±0.45	< 0.20	0.80±0.54	0.18±0.02	6.8±0.0	18.2	30.8	87.4
L-Rhamnose	1.00	42.5±1.8	1.11±0.08	5.84±0.15	1.15±0.06	7.06±0.31	< 0.20	2.46±0.02	0.16±0.01	6.9±0.2	6.0	35.3	96.0
L-Rhamnose	2.12	50.0±2.2	3.86±0.58	5.52±0.13	2.28±0.02	6.35±0.48	< 0.20	4.29±0.41	0.16±0.02	6.8±0.1	19.3	31.8	101.5
L-Rhamnose	5.62	52.5±1.3	1.68±0.35	6.66±0.35	0.81±0.11	9.66±2.29	< 0.20	9.67±1.79	0.15±0.01	6.8±0.2	8.4	48.3	93.4

**Table 5:** Effect of initial phosphate concentration on fermentation of selected sugar (10 mM) by *Clostridium* strain AK1 (DSM 18778). Values represent the average of triplicates with standard deviation shown as error bars.

Substrate	Phosphate concentration (mM)	Sugar consumed (%)	Analyte (mmol L^-1^)												
			Ethanol	Acetate	Butyrate	1,2-PD	Lactate	H_2_	OD_600 nm_		pH	Ethanol Yield (%)		1,2-PD Yield (%)	Carbon balance
D-Glucose	0	75.1±2.1	7.52±1.13	3.14±0.12	< 0.20	< 0.20	3.16±0.40	3.16±0.70	0.06±0.02		6.7±0.1	37.6		0.0	95.0
D-Glucose	0.01	90.1±1.0	11.01± 0.36	3.13±1.45	< 0.20	< 0.20	2.93±1.69	4.35±0.81	0.07±0.02		6.6±0.2	55.1		0.0	96.4
D-Glucose	0.05	96.9±0.3	12.59±1.01	4.15±0.46	< 0.20	< 0.20	2.61±0.84	6.08±1.28	0.09±0.02		6.5±0.1	63.0		0.0	101.0
D-Glucose	0.10	98.8±0.6	12.84±1.60	4.28±0.60	< 0.20	< 0.20	2.79±0.59	5.21±0.35	0.09±0.02		6.6±0.2	64.2		0.0	101.4
D-Glucose	0.25	95.0±0.7	12.24±1.27	4.03±0.29	< 0.20	< 0.20	2.54±0.52	6.06±1.01	0.09±0.03		6.5±0.1	61.2		0.0	100.5
D-Glucose	0.50	97.9±2.0	12.50±1.33	4.11±0.43	< 0.20	< 0.20	3.21±0.47	6.26±1.32	0.09±0.02		6.4±0.2	62.5		0.0	102.4
D-Glucose	1	93.2±1.2	10.94±0.99	4.12±0.25	< 0.20	< 0.20	3.18±1.00	5.62±1.11	0.090.03		6.3±0.1	54.7		0.0	99.5
D-Glucose	5	95.0±1.2	10.89±1.01	4.16±0.55	< 0.20	< 0.20	3.31±1.25	6.93±1.93	0.10±0.02		6.3±0.1	54.4		0.0	97.9
D-Glucose	15	90.2±0.5	11.23±0.97	4.21±0.44	< 0.20	< 0.20	2.06±0.12	6.89±0.42	0.19±0.10		6.2±0.1	56.2		0.0	98.8
D-Glucose	25	61.0±3.4	5.92±0.86	3.48±0.08	< 0.20	< 0.20	2.14±0.73	4.48±1.26	0.27±0.06		6.0±0.2	29.6		0.0	97.3
L-Rhamnose	0	56.0±2.6	4.09±0.33	3.66±0.18	0.73±0.37	2.02±0.36	< 0.20	2.84±0.53	0.05±0.01		6.9±0.0	40.1		20.2	100.2
L-Rhamnose	0.01	61.2±2.0	3.61±0.19	3.12±0.34	1.18±0.03	1.91±0.43	< 0.20	2.43±0.32	0.06±0.01		6.9±0.1	36.1		19.1	90.1
L-Rhamnose	0.05	59.3±1.3	3.69±0.24	2.99±0.17	1.31±0.05	1.78±0.38	< 0.20	2.67±0.33	0.06±0.01		7.0±0.2	36.9		17.8	93.8
L-Rhamnose	0.10	54.2±1.7	3.46±0.13	2.82±0.16	1.23±0.07	1.56±0.22	< 0.20	2.06±0.26	0.06±0.01		6.9±0.2	34.6		15.6	95.4
L-Rhamnose	0.25	53.4±2.1	3.41±0.12	2.87±0.09	1.12±0.13	1.66±0.08	< 0.20	2.02±0.19	0.06±0.01		6.8±0.1	34.1		16.6	95.9
L-Rhamnose	0.50	57.1±2.5	3.58±0.05	3.56±0.16	0.91±0.37	2.19±0.65	< 0.20	2.56±0.33	0.05±0.01		6.8±0.1	35.8		21.9	97.8
L-Rhamnose	1	53.8±1.1	3.25±0.27	3.08±0.09	1.07±0.08	1.89±0.40	< 0.20	2.28±0.43	0.06±0.01		6.8±0.2	32.5		18.9	95.8
L-Rhamnose	5	53.0±1.5	3.38±0.43	3.01±0.32	0.99±0.01	1.84±0.45	< 0.20	2.07±0.31	0.06±0.01		6.7±0.1	33.8		18.4	96.3
L-Rhamnose	15	57.3±1.2	3.77±0.15	3.35±0.24	1.05±0.07	2.05±0.60	< 0.20	2.36±0.11	0.03±0.003		6.7±0.1	37.7		20.5	98.9
L-Rhamnose	25	43.4±2.5	2.70±0.30	2.22±0.77	1.06±0.05	1.28±0.18	< 0.20	1.96±0.16	0.22±0.07		6.8±0.0	27.0		12.8	96.7

## 2 Experimental design, materials and methods

### 2.1 General methods

Yeast extract was obtained from Difco. L-Fucose was obtained from Dextra (Reading, UK). All other reagents were obtained from Sigma-Aldrich. Nitrogen gas was acquired from AGA and contained less than 5 ppm O_2_.

### 2.2 Microorganism and cultivation

*Clostridium* strain AK1 (DSM 18778) was isolated from the laboratory of the authors as described in [2] and reobtained from Deutsche Sammlung von Mikroorganismen und Zellkulturen (DSMZ). Strain AK1 was cultivated in serum bottles using the in Basal Mineral (BM) medium prepared as previously described [3]; BM consisted of (per liter): NaH_2_PO_4_·2H_2_O 3.04 g, Na_2_HPO_4_·2H_2_O 5.43 g, NH_4_Cl 0.3 g, NaCl 0.3 g, CaCl_2_·2H_2_O 0.11 g, MgCl_2_ × 6H_2_O 0.1 g, yeast extract 2.0 g, resazurin 1 mg, trace element solution 1 mL, vitamin solution (DSM141) 1 mL, and NaHCO_3_ 0.8 g. The trace element solution consisted of the following on a per liter basis: FeCl_2_ × 4 H_2_O 2.0 g, EDTA 0.5 g, CuCl_2_ 0.03 g, H_3_BO_3_, ZnCl_2_, MnCl_2_ × 4 H_2_O, (NH_4_)Mo_7_O_24_, AlCl_3_, CoCl_2_ × 6 H_2_O, NiCl_2_, and 0.05 g, Na_2_S × 9 H_2_O 0.3 g, and 1 mL of concentrated HCl. The carbon source concertation 20 mM unless stated otherwise. The medium was prepared by adding the buffer to distilled water containing resarzurin and boiled for 10–15 min until pink and cooled to ambient temperature under a stream of nitrogen (< 5 ppm O_2_). The mixture was then transferred to serum bottles using the Hungate technique [4], [5] and autoclaved for 60 min. All other components of the medium were added separately through filter (0.45 µm) sterilized solutions. All experiments were conducted at 50 °C and at pH of 7.0 with a liquid-gas (L-G) ratio of 1:1 unless specifically stated otherwise. In all cases, growth experiments were performed in triplicate without agitation.

### 2.3 Effect of initial substrate concentration

To investigate the effect of different initial glucose and rhamnose concentrations on growth the strain was cultivated at 10, 20, 40 and 60 mM of each sugar.

### 2.4 Effect of pH on fermentation end products

To investigate the effect of pH on growth the strain was cultivated at pH ranging from pH 4.0 to 9.0 with 0.5 pH unit intervals. End products were determined after 5 days of incubation.

### 2.5 Effect of temperature on fermentation end products

To investigate the effect of temperature on growth the strain was cultivated at 35 °C to 65 °C in 5 °C intervals. End products were determined after 5 days of incubation.

### 2.6 Effect of liquid-gas phase ratio

Strain AK 1 was cultivated in serum bottles (57 mL nominal volume) were filled with either 4.5, 13.4, 26.5, 36.0, or 45.0 mL of BM medium to give L-G values of 0.09, 0.34, 1.00, 2.12, and 5.62, respectively.

### 2.7 Effect of initial phosphate concentration

Phosphate-free yeast extract was prepared according to the method described by [6] and used for the preparation of phosphate-free BM. The phosphate concentration of the resultant yeast extracted was verified colorimetrically and was below the limit of detection of the assay. Strain AK1 was cultivated on 10 mM of either D-glucose or L-rhamnose with phosphate added from syringe-filtered stock bottles. Phosphate concentrations ranging from 0 (control) and 0.01 mM to 25 mM were investigated.

### 2.8 Analytical methods

Hydrogen, volatile fatty acids and alcohols were quantified by gas chromatography as described earlier [2]. D-Glucose was quantified using the anthrone method [7] with the modifications described by previously [8]. Methylpentoses and 1,2-PD were analysed using colorimetric methods [9], [10] modified for microplates as described earlier [8]. Lactic acid was quantified using the method of [11] with modification; Briefly, 50 µL of sample was placed in a 1.5 mL Eppendorf tube, followed by the addition of 300 µL of concentrated sulfuric acid and incubated in a water bath (100 °C) for 10 min. After cooling to RT, 5 µL of 4% (w/v) CuSO_4_ reagent were added followed by 10 µL of 1.5% (w/v) *p*-phenylphenol in 95% (v/v) ethanol. The mixture was vortexed and allowed to incubate at room temperature for 30 minutes. 300 µL of sample was then transferred into a microtiter plate and read at 570 nm against a water blank. Phosphate concentrations were determined spectrophotometrically according to Olsen and Summers [12] in microtiter plates read in a Bioscreen C.

Optical density was measured at 600 nm with a Shimadzue UV-1800 UV-Visible spectrophotometer with cuvetted (*l* = 1 cm) against a water blank.
