# Supplementary material for: Dataset describing ethanol and 1,2-propanediol production by a stenothermal moderately thermophilic anaerobe, Clostridium strain AK1
Source: Data Brief. 2018 Aug 30;20:649–57. doi: 10.1016/j.dib.2018.08.088 (PMC6127976; doi:10.1016/j.dib.2018.08.088)
Supplement: Supplementary file 1 — Supplementary material [file mmc1.docx]

Conflict of interest.

The authors claim there are no conflict of interest concerning the material presented in the submission of the manuscript „Dataset Describing Ethanol and 1,2-Propanediol Production by a Stenothermal Moderately Thermophilic Anaerobe, *Clostridium* strain AK1

“.
